# Effects of modified trans-tibial versus trans-portal technique on stress patterns around the femoral tunnel in anatomical single-bundle ACL reconstruction with different knee flexion angles using finite element analysis

**DOI:** 10.1186/s12891-022-05713-y

**Published:** 2022-08-08

**Authors:** Hyun-Soo Moon, Si Young Song, Ji Ung Oh, Young-Jin Seo

**Affiliations:** 1 Department of Orthopedic Surgery, Hallym University Sacred Heart Hospital, Anyang, Gyeonggi-do Republic of Korea; 2 Department of Orthopedic Surgery, Hallym University Dongtan Sacred Heart Hospital, 7, Keunjaebong-gil, Hwaseong, Gyeonggi-do Republic of Korea

**Keywords:** Anterior cruciate ligament (ACL), Trans-portal, Trans-tibial, Single-bundle, Reconstruction, Stress

## Abstract

**Background:**

It is unclear whether different anterior cruciate ligament (ACL) graft trajectories in the distal femur would have different effects on stress generated within the distal femur around the femoral tunnel during knee motion. Thus, the purpose of this study was to determine differences in stress patterns around the femoral tunnel created by trans-portal (TP) vs. modified trans-tibial (TT) technique in anatomical ACL reconstruction at different knee flexion angles.

**Methods:**

Twelve male subjects’ right knees were scanned with a high-resolution computed tomography (CT) scanner (slice thickness: 1 mm) at four different knee flexion angles (0°, 45°, 90°, and 135°). Three-dimensional (3D) models of these four different flexion angles were created and manipulated with several modelling programs. For the TP group, the virtual femoral tunnelling procedure was performed in a 135° flexion model from the low far anteromedial (AM) portal. For the modified TT group, the same knee models were drilled through the modified TT technique at 90° of flexion separately. Virtual grafts under tension of 40 N were put into corresponding bone tunnel and fixed at the outer aperture of femoral tunnels to simulate the suspensory fixation, followed by fixation of the grafts at the middle of tibial tunnels in the 0° knee flexion models. Finally, the models were exported to a finite element analysis package and analysed using ABAQUS/Explicit code (ABAQUS, USA) to monitor the stress occurring at the node where stress distribution occurred most significantly in the femoral bone around the bone tunnel.

**Results:**

In general, both groups showed a high stress distribution in bony structures around inner and outer orifices of the femoral tunnel. Mean maximal stresses occurring at the lateral femoral condyle around the inner orifice of the femoral tunnel in the TP group were found to be significantly greater than those in the modified TT group at all flexion angles except 90° of flexion. Mean maximal stresses monitored around the outer orifice of the femoral tunnel in the TP group were also significantly greater than those in the modified TT group at all flexion angles.

**Conclusions:**

Different tunnelling technologies could yield different stress patterns in the lateral femoral condyle around the femoral tunnel. During knee motion, higher stresses were noticed in the TP group than in the modified TT group, especially around inner and outer orifices of the tunnel. Position of the tunnel after reconstruction with the TP technique can have a greater effect on the stress increase in the femur compared to that with the modified TT technique.

## Introduction

Based on numerous biomechanical studies regarding anterior cruciate ligament (ACL) reconstruction, bone tunnel created within the native footprint has been advocated to improve rotational stability [1–3]. To achieve femoral sockets which lies in the native footprint, numerous studies have demonstrated technical advances of tans-portal (TP) and modified trans-tibial (TT) ACL reconstruction. These two techniques are currently popular among surgeons to perform anatomical ACL reconstruction. An ACL graft with a modified TT tunnel technique lies in different trajectories in the distal femur compared to that with a TP technique because of different femoral tunnel centers and tunnel directions. Some authors have shown that a modified TT technique can be performed utilizing an oblique shallow tibia tunnel to enable the femoral tunnel to lie within the original ACL footprint in the anteromedial (AM) bundle region [4, 5]. Whereas, TP femoral tunnel could be created independently from the tibial tunnel and placed at the “intuitive center” of the femoral attachment, which lies between the AM and posterolateral (PL) footprint [6–8].

Different trajectories of the ACL graft could yield different tunnel parameters including length of the femoral tunnel and femoral graft bending angle [9–13]. Different tunnel parameters are consequently associated with different stress patterns around the femoral tunnel [14–18]. Hoshino et al. in their cadaveric study, reported that different mechanical stress around the femoral tunnel was exhibited according to different directions [14]. Recent Finite element studies have demonstrated that the contact stress arising at the interface between the graft and the surrounding bony structures was influenced by different femoral tunnel positions, and changes in the reaction force and maximal stress of the graft was also affected by knee motion [15, 18].

Stresses arising in the bony structure due to interaction between the ACL graft and the bone tunnel have not been well investigated yet. Increased contact pressure around the femoral tunnel may erode tunnel aperture, resulting in tunnel enlargement [19]. Although tunnel expansion might not produce a detectable advantage in clinical outcome measures, femoral tunnel widening might be associated with increased anterior joint laxity [20]. Furthermore, the tunnel expansion phenomenon may subsequently compromise ACL revision surgery. Therefore, knowledge of stress patterns within the distal femur adjacent to the bone tunnel during knee motion could provide a possible explanation for the postoperative tunnel widening phenomenon and serve as a useful basis for an improved outcome after ACL reconstruction.

The present study hypothesizes that different ACL graft trajectories in the distal femur would have different effects on the stress generated within the distal femur around the femoral tunnel during knee motion. To test this hypothesis, anatomically detailed three-dimensional (3D) knee model was reconstructed and virtual ACL reconstruction was performed using several modelling programs. The 3D-model was then evaluated using a finite element analysis. The purpose of this study was to determine differences in stress generated within the distal femur around the bone tunnel created by TP vs. TT technique in anatomical ACL reconstruction at different knee flexion angles.

## Material and methods

### Overall experimental algorithm

3D right knee models of 12 subjects were created from computed tomography (CT) data and used for analysis after virtual ACL reconstruction using finite element analysis. This study was approved by our Institutional Review Board. Informed consent was obtained from all subjects. Inclusion criteria were healthy adults who denied any form of knee pathologies. Subjects were excluded if they had previous injuries to their knees that required a visit to the hospital. Subjects were 12 males. Their mean age at the time of this study was 33.8 ± 7.5 (range, 23 - 49) years.

Digital Imaging and Communications in Medicine (DICOM) files obtained from CT scanning were exported into an image processing software (Amira, R 4.0 TGS, USA). A 3D knee model was then reconstructed by extracting and stacking bony regions from the acquired DICOM files. Virtual surgery was then performed on anatomically detailed 3D knee models using a special 3D data processing program (Rapidform 2006 INUS, Korea). Virtual single-bundle ACL reconstruction was performed using either the TP technique (TP group) or the modified TT technique (modified TT group). Each group included all 12 subjects because the 3D computational process we adopted enabled us to perform different virtual surgeries on the same knee model, thus reducing effects of inter-subject variation.

The tibia and femur in 3D knee models were meshed with 4-node tetrahedron elements. Reconstructed ACLs were meshed with 8-node hexagon elements using Hyperworks (Altair engineering, USA). Because of geometrical complexity of the bony structure, the bone was generated as tetrahedrons, which could more easily capture the irregular shape [21].

Heterogeneous material properties were assigned on an element specific basis. Finally, the model was exported to a finite element analysis package and analysed using an ABAQUS/Explicit code (ABAQUS, USA) to monitor the stress occurring at the node where stress distribution occurred the most significantly in the the femoral bone around the bone tunnel.

### Simulation of ACL reconstruction

**Fig. 1:**
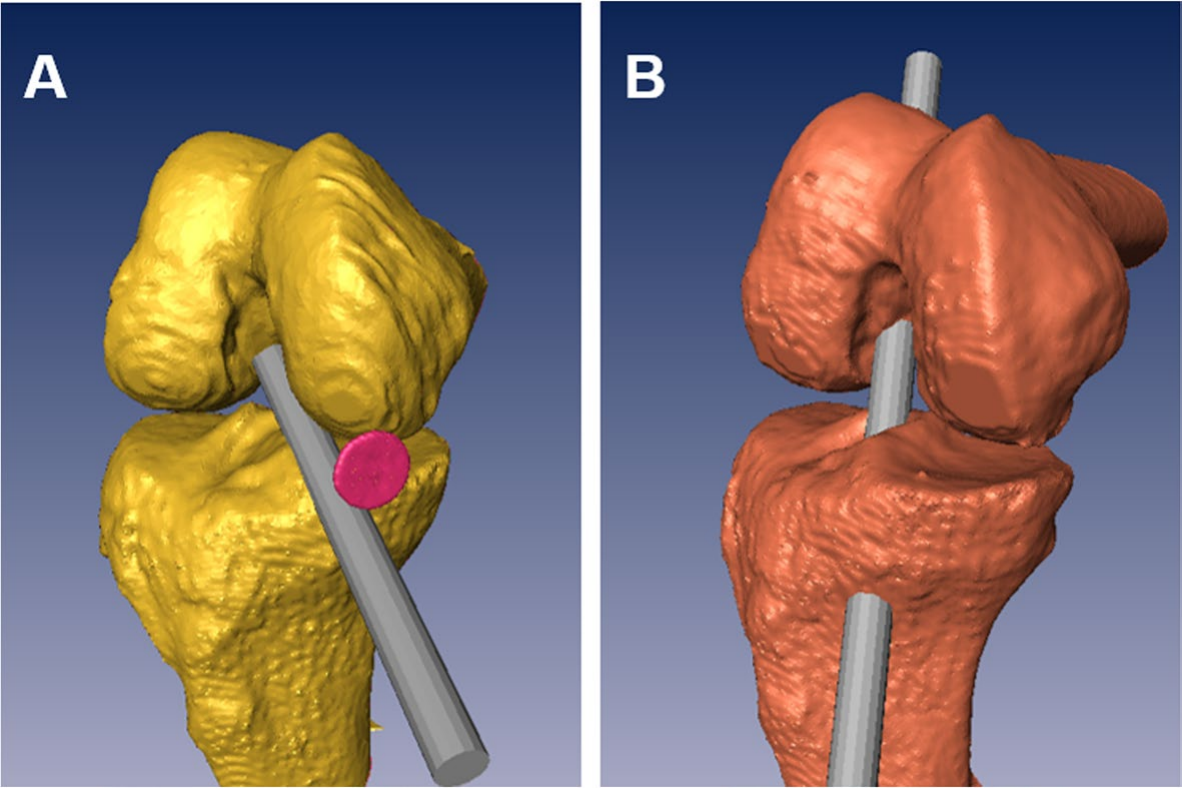
Computational simulation of femoral tunnel drilling. The femoral tunnel of 10 mm diameter was created in 135° flexion model from the low far anteromedial portal which was previously marked with a coin during CT scanning (TP group, **A**). The same knee models were then drilled through the modified TT technique (modified TT group, **B**) with the same diameter at 90° of flexion separately. TP: trans-portal, TT: trans-tibial

To simulate an anatomical single-bundle ACL reconstruction using the TP technique, the femoral tunnel of 10 mm diameter was created in 135° flexion model from the low far anteromedial portal which was previously marked with a coin during CT scanning (TP group). The same knee models were then drilled with the modified TT technique (TT group) using the same diameter at 90° of flexion separately (Fig. 1).

In particular, in the TP group, the optimal femoral tunnel was verified in 3D knee models, in which center of the femoral tunnel was located posterior to the lateral intercondylar ridge. And the center of the femoral tunnel was also placed in the bifurcate ridge, which seperates the AM and PL footprint [22–24]. The center of the tibial tunnel was placed according to the information of the tibial bony landmarks based on a previous cadaveric study [25, 26]. Based on these observations, the center of the tibial tunnel was established on the 3D model of the tibia.

In the TT group, the starting point for the tibial tunnel was set at 4 ~ 5 cm distal to the joint line and 2 ~ 3 cm posteromedial to the tibial tuberosity [27]. The virtual reamer was then introduced from the extra-articular tibial entry point into the tibial footprint at a point 1-2 mm medial to the center of tibial spine, a location similar to the tibial tunnel center created during the TP group, and directed as close as possible towards the center point within the femoral footprint. If the aiming point toward femoral footprint was placed in too superior position, varus tilting and internal or external rotation of the tibia up to 5° were applied to adjust directional angle toward the femoral footprint according to previous literature describing the modified TT technique [27–29]. To decrease error, the center point of the tunnel was determined by a senior surgeon (SYJ). All virtual tunnelling processes were done with a special software (Rapidform 2006 INUS, Korea).

Computational processing for knee flexion was adopted from our previous reports regarding computational 3D image analysis [30, 31]. First, discrete tibiae at 0°, 45°, 90° and 135° of each flexion knee model were superimposed. In the TP group, the femur including femoral tunnel in 135° flexed knee model was then moved to superimpose femurs in other angles of flexion and replace them. As a result, identical tunnel was located at each flexed model. In the TT group, the femur in 0°, 45°, and 135° of flexed knee model was replaced with the femur in 90° flexed model in which desired femoral tunnel was created in the same manner as in the TP group. The superimposition performance was ensured by the 3 D coordinate system in the special software (Rapidform 2006 INUS, Korea).

The material characteristics of the reconstructed ACL are expressed as a mathematical equation, characterized by a strain energy potential function such as in an Ogden model [21]. The stress-strain relationship and curve fitting was performed using the hyperelastic material model [21, 32].

The bony structure was assumed to be isotropic linear elastic, which is also adequate for the study of stress and strain. The isotropic Poisson’s ratio and the Young’s modulus were adopted from data available in the literature [33, 34].

Grafts were fixed at the outer aperture of femoral tunnels to simulate the suspensory fixation. Although cortical suspensory fixation device itself was not modeled, the technology of the graft fixation chosen in this study was determined based on a study protocol reporting FEM performance of a 3D knee model [15]. A set pretension of 40 N was then applied to grafts in the 0° knee flexion model, followed by fixation of grafts at the middle of tibial tunnels, where a tied interaction between the bone tunnel and the graft was determined. Bone-ligament contacts were modelled using penalty formulation. Frictional coefficient was set to be 0.1 [21].

### Measurement methods

Femoral tunnel measurement was performed with use of a true side view of the 3D model. A rectangular grid was aligned with intercondylar notch roof based on radiographic quadrant method as previously described by Bernard et al. [23, 35] and explained in detail using 3D CT models by Forsythe et al. [23, 35] Measurements were then performed in terms of percent distances of the grid from the posterior border of the lateral femoral condyle to the tunnel center (in the posterior-to-anterior (deep/shallow) directions) and from the intercondylar notch roof to the tunnel center (in the proximal-to-distal (high/low) directions).

**Fig. 2:**
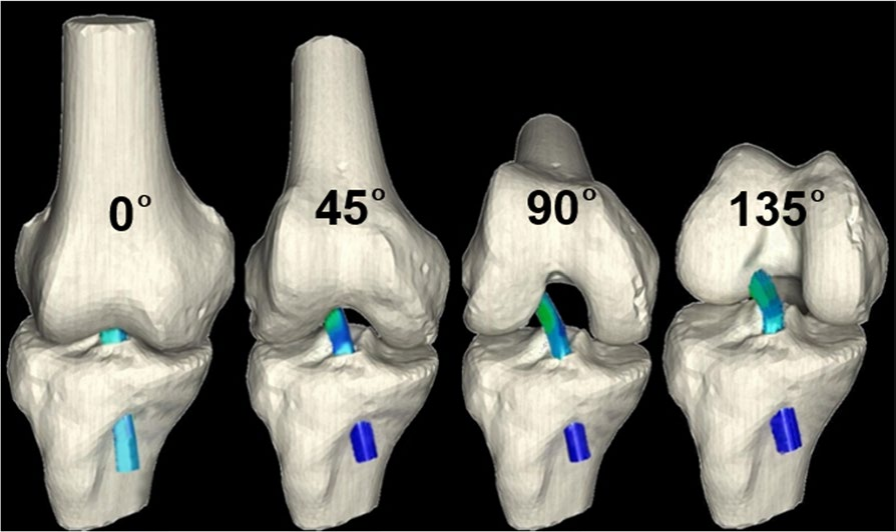
A sequential flexion of ACL reconstructed knee model was reproduced at four different angles (0°, 45°, 90° and 135°) as the femur was moved to each flexed position according to a 3D coordinate system based on the fixed tibia. Meanwhile, the stress concentration and its maximum value were monitored in the lateral femoral condyle around the femoral tunnel

A sequential flexion of finite element model (ACL reconstructed knee model) was reproduced at four different angles (0°, 45°, 90° and 135°) as the femur was moved to each flexed position according to a 3D coordinate system based on the fixed tibia. Meanwhile, stress concentration and its maximum value were monitored in the lateral femoral condyle of the femur around the femoral tunnel (Fig. 2).

### Statistical analysis

A priori power analysis was performed using power calculation tool of G*Power for Student’s t-test (v3.1.2) to determine the validity of the number of subjects necessary to distinguish significant differences in maximal stress generated within the surrounding bone structure near the tunnel after TP and modified TT ACL reconstruction. Means of difference in stress generated were defined as 1.3 MPa based on previous literature [14].

A sample size analysis with a power of 80% and an alpha of 0.05 showed that 10 subjects in each group were required. Thus, 12 subjects in each group were assumed to be sufficient for the statistical analyses in this study.

Differences in femoral tunnel position between the two groups were compared using Student’s t-test. Inter- and intra-observer reliabilities of each measurement are presented with intraclass correlation coefficients (ICC). To access ICC, two observers evaluated each measurement twice with a one-week interval. Differences in the maximal stress concentration at different knee flexion angles were statistically analysed using one-way analysis of variance (ANOVA) with the Tukey honestly significant difference (HSD) test for pair-wise comparisons. Comparisons of the maximum stress within the femoral bone around the femoral tunnel between two groups at four different angles were performed using Student’s t-test. The level of significance was set at *P* < 0.05. All statistical analyses were performed using Statistical Package for Social Science (Version 26; SPSS Inc., Chicago, IL, USA).

## Results

### Femoral tunnel location

The femoral tunnel for the TP group was located 29.2 ± 3.4% in posterior-to-anterior (deep/shallow) direction and 35.1 ± 3.9% in proximal-to-distal (high/low) direction. The femoral tunnel of the modified TT group was located 38.6 ± 5.3% in posterior-to-anterior (deep/shallow) direction and 29.5 ± 3.7% in proximal-to-distal (high/low) direction. The difference in mean femoral tunnel locations expressed as percentage distance between the two groups was significantly different (posterior-to-anterior: *P* < 0.001, proximal-to-distal: *P* = 0.002). In other words, the femoral tunnel of the modified TT technique was placed in more anterior and proximal location than that of the TP technique. ICC values for inter- and intra-observer reliabilities for the femoral tunnel position in posterior-to anterior directions (0.85 and 0.87) and in proximal-to distal directions (0.80 and 0.89) were considered to be satisfactory.

### Stress patterns in the femoral bone around the tunnel

**Fig. 3:**
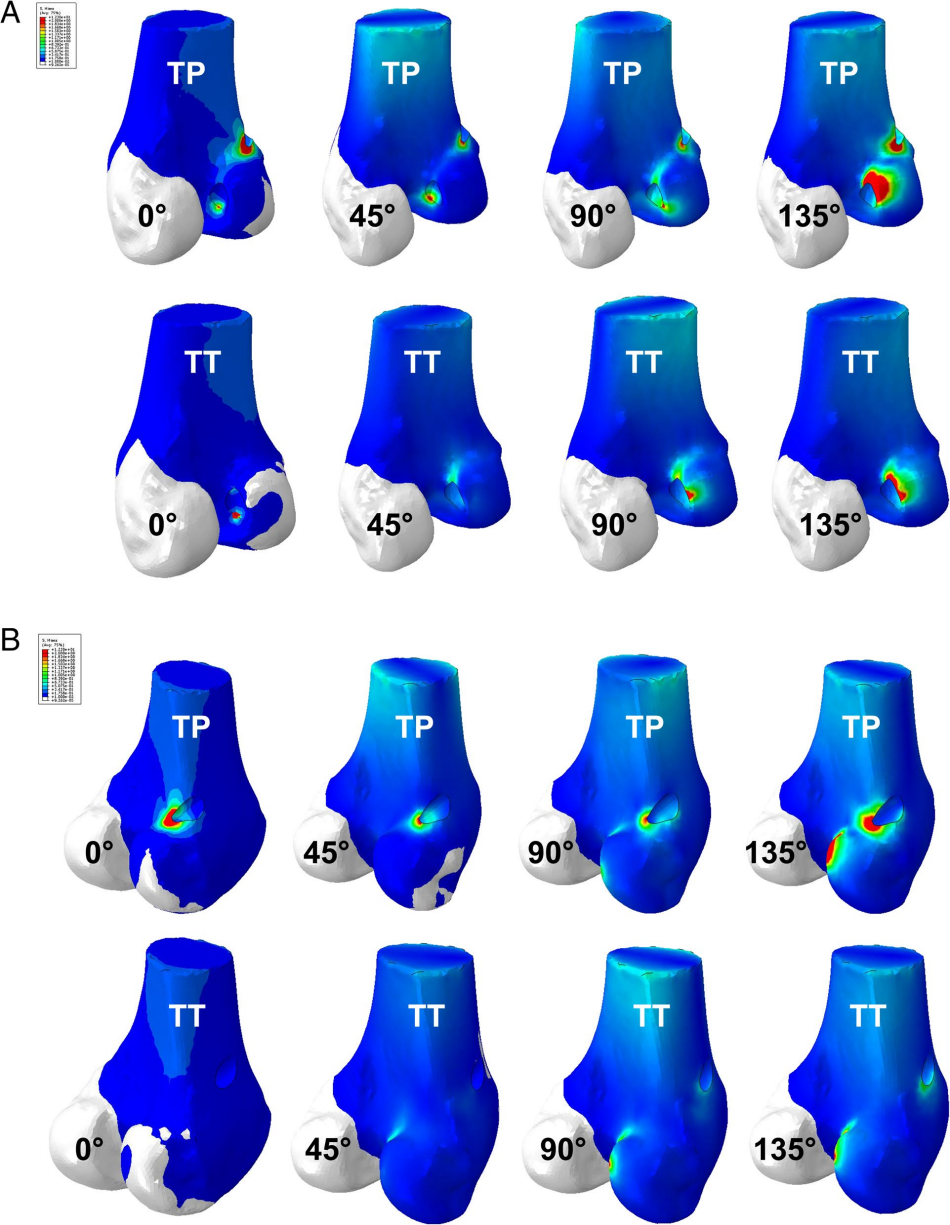
Patterns of stress distribution at different flexion angles in the knee models with the TP or the modified TT technique. At 0° of flexion, the highest stress was seen on the anterior margin of the inner orifice of the tunnel in both groups. At 90° and 135° of flexion, the site of the highest stress moved to the posterior part of the inner orifice (**A**). The maximal stress concentration was also monitored around the outer orifice of the tunnel where the tendon was fixated in both groups (**B**). These figures are from a single representative specimen (TP group: upper column, modified TT group: lower column)

We found that there were common trends in stress patterns around the inner orifice of femoral tunnel regardless of drilling method. At 0° of flexion, the highest stress was seen on the anterior margin of the inner orifice of the tunnel where the contact between the bone and the graft occurred in both group. At 90° and 135° of flexion, the site of the highest stress moved to the posterior part of the inner orifice. Meanwhile, high stress concentration was also monitored around the outer orifice of the tunnel where the tendon was fixated in both groups. In this area, the stress concentration was more predominant in the TP group than in the modified TT group at all flexion angles. Overall stress patterns are shown in Fig. 3.

### Comparison of the maximal stress

In general, the maximal stresses occurring at the lateral femoral condyle around the inner orifice of the femoral tunnel in the TP group were found to be significantly greater than those in the modified TT group at all flexion angles except 90° of flexion (at 0°, 45°, 90° and 135°: *P* = 0.015, *P* = 0.027, *P* = 0.404, and *P* = 0.022, respectively). The maximal stresses monitored around the outer orifice of the femoral tunnel in the TP group also showed significantly greater values than those in the modified TT group at all flexion angles (at 0°, 45°, 90° and 135°: *P* < 0.001, *P* = 0.031, *P* = 0.011, and *P* = 0.005, respectively).

In the TP group, the mean maximum stress value of 4.3 ± 0.9 MPa was monitored around the inner orifice at 135° of flexion, which was significantly greater than those at other flexion angles (all *P* < 0.001). Whereas in the TT group, as the knee flexion increased beyond 45° of knee flexion, the mean maximal stress around the inner orifice was significantly greater than that at 0° or 45° of flexion (0° vs. 90°, 0° vs. 135°, and 45° vs. 135°: all *P* < 0.001; 45° vs. 90°: *P* = 0.002).

**Fig. 4:**
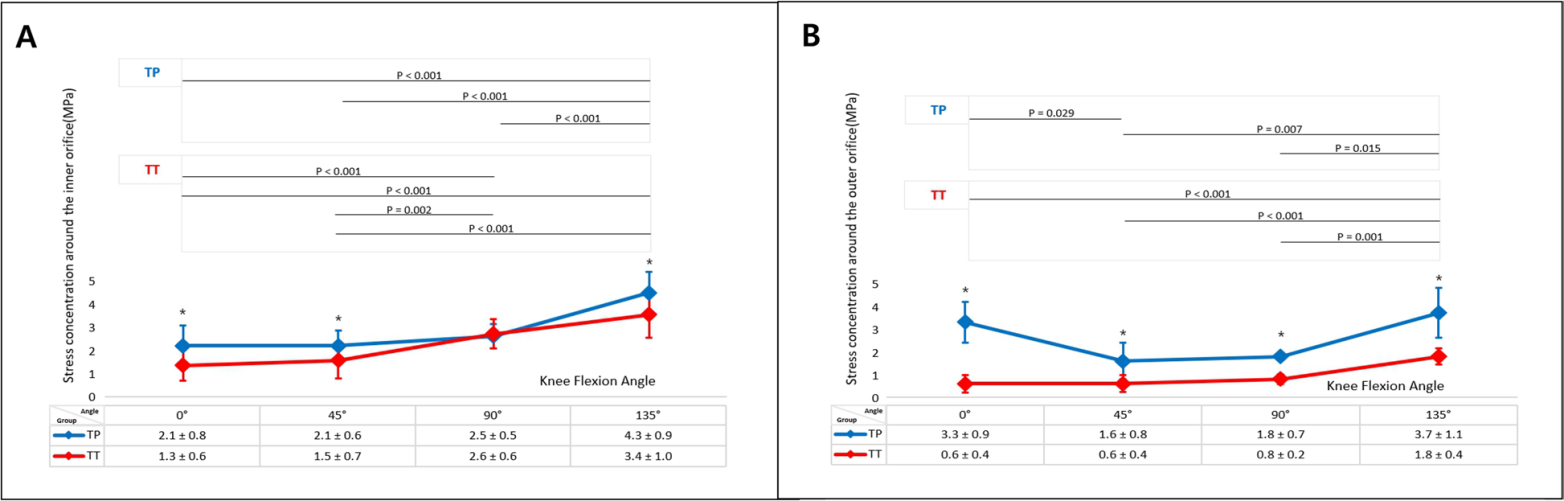
Maximal stresses monitored around the inner orifice in the TP group were found to be significantly greater than those in the modified TT group at all flexion angles except 90° of flexion (**A**). Those around the outer orifice in the TP group also generated significantly greater values than those in the modified TT group at all flexion angles (**B**). * Denoted statistical significancy (*P* < 0.05). Maximal stress values in each group at discrete flexion angles were presented as mean ± standard deviation (MPa). Significance lines with *p*-values represented that the mean maximal stresses at discrete knee flexion angles are significantly different from one another in each group. TP: trans-portal, TT: trans-tibial

In terms of maximal stress generated around the outer orifice, in the TP group, the mean maximal stress value of 3.3 ± 0.9 MPa at 0° significantly decreased at 45° (*P* = 0.029). it remained constant between 45° and 90° flexion interval with no significant difference (*P* = 0.792), followed by significant increase as knee flexion increased from 90° to 135° flexion (*P* = 0.015). In the TT group, the mean maximum stress value of 1.8 ± 0.4 MPa was monitored around the outer orifice at 135° of flexion, which was significantly greater than those at other flexion angles (0° vs. 135°, 45° vs. 135°: all *P* < 0.001; 90° vs. 135°: *P* = 0.001). The relationship between the maximal stress value and the knee flexion angle is shown in Fig. 4.

## Discussion

This study presented a finite element model allowing the monitoring of stress concentration around the femoral tunnel at discrete knee flexion angles using images from living subjects. Most studies regarding effects of TP and TT techniques on the biomechanics of an ACL-reconstructed knee have been conducted using cadavers. Stress patterns around the femoral tunnel with respect to different drilling methods, especially those based on in-vivo knee kinematics, have been rarely reported.

The most principal finding of our study was that the maximum value of stress monitored at bony structures around the femoral tunnel was found to be higher in the TP group than in the modified TT group. We found the highest stress around the inner orifice where the contact between the bone and the graft occurred. Hoshino et al. [14] have demonstrated stress patterns around the femoral tunnel following ACL reconstruction with a hamstring graft and found that the distal region has the largest stress at full knee extension, similar to our findings.

It is now generally agreed that the femoral tunnel which is placed lower in the notch could provide better restoration of native knee biomechanics, especially in terms of restoration of rotational instability [9, 36–38]. To achieve more horizontal graft, some surgeons have suggested the use of a TP technique for femoral tunnel drilling [10, 39–42]. However, some surgeons have demonstrated that a modified TT tunnel technique with the tibial tunnel starting from a medial position could provide a femoral tunnel closer to the anatomic position [43]. A recent CT based 3-D modelling study showed that inner orifice of the femoral tunnel with the modified TT technique was positioned at more anterior and proximal location compared to coordinates of the native ACL center [44]. Our results also showed that the center of the tunnel in the modified TT group was placed at more anterior and proximal position than that in the TP group.

Different tunnelling technologies could yield different directions of the tunnel socket in the distal femur. Different tunnel-graft bending angles could produce different contact stress at the graft-bone interface [15]. Furthermore, among various factors that can potentially affect femoral tunnel widening phenomenon after ACL reconstruction, the position of the tunnel could be regarded as a possible factor [45].

The higher stress pattern around the femoral tunnel in the TP group than that in the modified TT group shown in this study might be due to a more acute graft-tunnel angle produced by the TP technique [16]. Our results also reinforce results of a previous cadaveric study by Segawa et al. showing that acute femoral tunnel could increase the mechanical stress in the femoral tunnel [19]. They monitored maximum contact pressure at the anterior part of the tunnel with the knee in full extension and at the posterior portion with deep knee flexion [19], which is partly in agreement with our results.

**Fig. 5:**
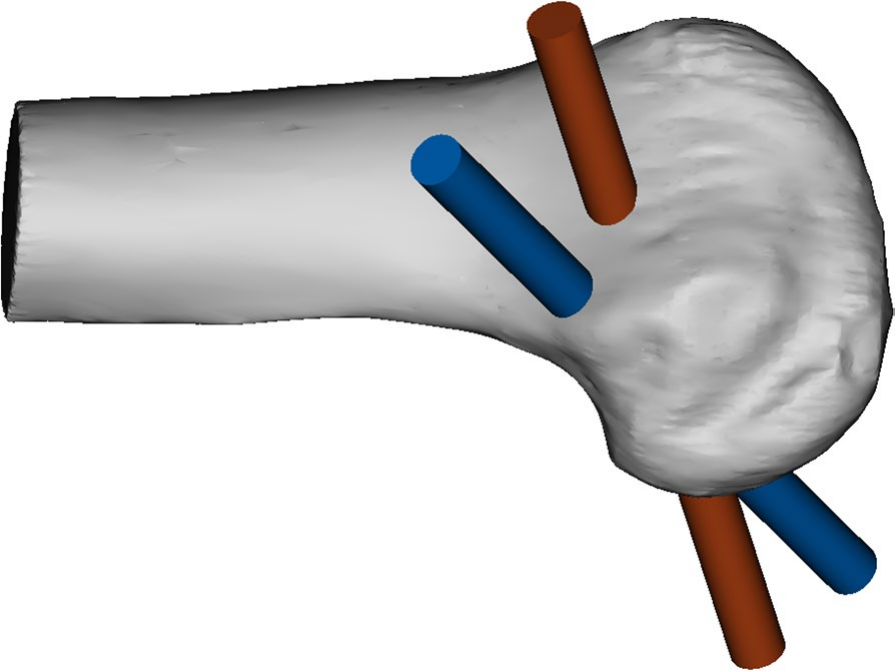
Outer exit of TP technique (blue) is more posteriorly placed that that of the modified TT technique (red). TP: trans-portal, TT: trans-tibial

Regarding the stress arising in the femoral tunnel exit, Smolinski et al. have performed a finite element model study and reported that different tunnel exit position after ACL femoral tunneling has different effects on femoral bone stress [17]. Although it is difficult to draw a direct comparison with our results due to different experimental scenarios, they demonstrated that stress concentration arising at the PL tunnel exit was greater than that at the AM tunnel exit, consistent with our results. That is, positioning outer exit of the femoral tunnel more posteriorly can increase the stress concentration at the exit. In general, outer exit of the TP technique was more posteriorly placed that that of modified TT technique in our study (Fig. 5).

Results of this study are clinically relevant because our data demonstrating changes in bone stress patterns generated by different tunnel locations could provide surgeons with relevant information to help them select the ACL femoral tunneling scenario.

However, results and clinical relevance of this study should be carefully interpreted in light of its several limitations. First, we did not plot or compare graft bending angle at the tunnel orifice to show relationship between graft bending angle and the stress monitored around the bone tunnel. However, several studies have demonstrated that the graft bending angle after the TP technique could be more acute than that after the TT technique, resulting in potential increase in mechanical stress around the bone tunnel orifice [10–13, 16, 28, 46]. Since recent meta-analaysis demonstrated that the comparison between the modified TT and the TP technique demonstrated no significance difference in the clinical outcomes including anteroposterior and rotational stabilities [47], other factors rather than the kinematic stability of postoperative knee need to be elucidated to determine the optimal ACL reconstruction technique. Hence, we focused the stress patterns arising in the femoral bone, which were not well investigated before.

Second, only maximal stress in the lateral femoral condyle was monitored in this study. Although we assumed that increase in stress concentration at the margin of the tunnel may contribute to bony structural change around the tunnel, other factors have also been suggested as potential causes of tunnel expansion, such as a deprivation of the stress in the bone, resulting in bone resorption [48]. Differences in tunnel stress patterns including minimal stress generated at various sites in the lateral femoral condyle need to be elucidated in future studies. Third, we considered the graft and bone to be linear elastic, homogeneous, incompressible, and mechanically isotropic. Although these assumptions are rational in terms of numerical modelling [32, 49, 50], a more realistic representation of mechanical properties of the graft and bone need to be developed in future studies.

## Conclusions

Different tunnelling technologies could yield different stress patterns in the lateral femoral condyle around the femoral tunnel. Position of the tunnel after reconstruction with the TP technique can have a greater effect on the stress increase in the femur than that with a modified TT technique.

## Data Availability

The datasets used and analysed during the current study are available from the corresponding authors upon reasonable request.

## Abbreviations

ACL: Anterior cruciate ligament
TP: Trans-portal
TT: Trans-tibial
3D: Three dimensional
CT: Computed tomography
AM: Anteromedial
PL: Posterolateral
DICOM: Digital Imaging and Communications in Medicine
ICC: Intraclass correlation coefficients
ANOVA: One-way analysis of variance
